# Metacognitive Failure as a Feature of Those Holding Radical Beliefs

**DOI:** 10.1016/j.cub.2018.10.053

**Published:** 2018-12-17

**Authors:** Max Rollwage, Raymond J. Dolan, Stephen M. Fleming

**Affiliations:** 1 Wellcome Centre for Human Neuroimaging, University College London, London WC1N 3BG, UK; 2 Max Planck University College London Centre for Computational Psychiatry and Ageing Research, London WC1B 5EH, UK

**Keywords:** metacognition, radicalism, change of mind, confidence, post-decision evidence, politics, cognitive flexibility

## Abstract

Widening polarization about political, religious, and scientific issues threatens open societies, leading to entrenchment of beliefs, reduced mutual understanding, and a pervasive negativity surrounding the very idea of consensus [1, 2]. Such radicalization has been linked to systematic differences in the certainty with which people adhere to particular beliefs [3, 4, 5, 6]. However, the drivers of unjustified certainty in radicals are rarely considered from the perspective of models of metacognition, and it remains unknown whether radicals show alterations in confidence bias (a tendency to publicly espouse higher confidence), metacognitive sensitivity (insight into the correctness of one’s beliefs), or both [7]. Within two independent general population samples (n = 381 and n = 417), here we show that individuals holding radical beliefs (as measured by questionnaires about political attitudes) display a specific impairment in metacognitive sensitivity about low-level perceptual discrimination judgments. Specifically, more radical participants displayed less insight into the correctness of their choices and reduced updating of their confidence when presented with post-decision evidence. Our use of a simple perceptual decision task enables us to rule out effects of previous knowledge, task performance, and motivational factors underpinning differences in metacognition. Instead, our findings highlight a generic resistance to recognizing and revising incorrect beliefs as a potential driver of radicalization.

## Highlights

• Metacognition refers to the ability to reflect on our cognitive processes
• We investigated metacognitive features of radicalism in a low-level perceptual task
• Radical participants showed less insight into the accuracy of their decisions
• Radicals showed smaller confidence shifts in response to disconfirmatory evidence

## Teaser

Rollwage et al. examined whether radical beliefs are linked to alterations in metacognition in a low-level perceptual task. Radical participants—on both ends of the political spectrum—showed reduced insight into the correctness of their choices and less sensitivity to post-decision evidence, indicating a generic resistance to revising mistakes.

## Results and Discussion

An unjustified certainty in one’s beliefs is a characteristic common to those espousing radical beliefs [3, 4, 5, 6], and such overconfidence is observed for both political and non-political issues [3, 4, 6], implying a general cognitive bias in radicals. However, the underpinnings of radicals’ distorted confidence estimates remain unknown. In particular, one-shot measures of the discrepancy between performance and confidence are unable to disentangle the contributions of confidence bias (changes in an overall belief about performance, which may be affected by optimism [8] and mood [9]) from changes in metacognitive sensitivity (an ability to distinguish accurate from inaccurate performance; [7]).

This distinction may be particularly important as changes in metacognitive sensitivity may account for radicals’ reluctance to change their mind in the face of new evidence. Decision neuroscience has highlighted that metacognitive sensitivity depends on mechanisms that facilitate monitoring and revision of confidence in previous choices [10, 11]. This ability relies on specific neural circuitry in the prefrontal cortex [12] that promotes reflection on one’s performance and, even in the absence of explicit feedback, a realization that mistakes have been made [13, 14]. It has generally been assumed that a resistance of radicals to change their beliefs is due to social and motivational factors, such as the desire to maintain a positive self-image [15, 16, 17, 18], whereas the role of metacognitive capacities has received less attention. However, changes of mind depend not only on a motivation to change but also on a (metacognitive) capacity to realize that one’s beliefs are wrong.

By employing simple perceptual discrimination tasks, it is possible to precisely quantify metacognitive sensitivity—the extent to which people’s confidence judgments are sensitive to task performance—and to disentangle metacognitive sensitivity from overconfidence bias [7]. Such tasks provide an objectively correct answer (which is rarely the case for direct assays of political attitudes where the ground truth is often unknown or unavailable), thus enabling a precise, quantitative, and objective measure of metacognitive ability as well as a normative prediction for changes of confidence in light of new evidence. Moreover, the usage of a perceptual task makes it unlikely that participants have *a priori* vested interests in a particular decision outcome, thus diminishing any strong link to participants’ self-concept and providing an assay of the relationship between domain-general metacognitive abilities and radicalism. Here, we test a hypothesis that limitations in metacognitive sensitivity lead to a resistance to belief change, even when motivational factors are minimized, and that such metacognitive limitations are associated with the entrenched beliefs that are exemplified by radicals.

To typify a spectrum of radical views, we first conducted a separate online survey of 344 US participants (study 1) who completed questionnaires about political issues [4, 19, 20, 21, 22]. We included standard questionnaires about political orientation, voting behavior, attitudes toward specific political issues, intolerance of opposing political attitudes, belief rigidity, and (left- and right-wing) authoritarianism. These questionnaires were selected based on prior models of political radicalism as stemming from a combination of intolerance to others’ viewpoints, dogmatic and rigid beliefs, and authoritarianism, which represents adherence to in-group authorities and conventions, and aggression in relation to deviance from these norms [23, 24, 25]. However, we stress that radicalism is likely to reflect a general cognitive style that transcends the political domain—as exemplified by links between religious fundamentalism and increased dogmatism and authoritarianism [22, 26]—and instead refers to how one’s beliefs are held and acted upon [27].

**Figure 1:**
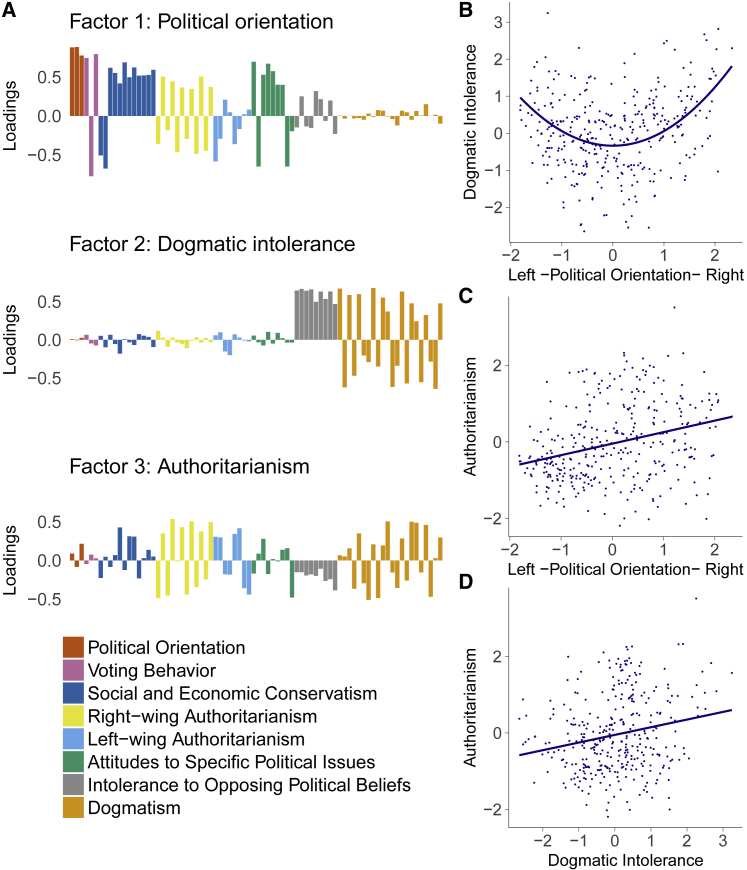
The Left and Right Extremes of the Political Spectrum Are Associated with Intolerant and Dogmatic World Views Data presented from study 1 (n = 344). (A) Using factor analysis, we investigated the underlying factor structure of multiple questionnaires about political issues. Three latent factors were identified and labeled “political orientation,” “dogmatic intolerance,” and “authoritarianism” according to the pattern of individual item loadings. Item loadings for each question (questionnaires indicated by different colors) are presented. (B–D) To investigate the relation between these constructs, scores on the three factors were extracted for each individual. (B) We observed a quadratic relationship between political orientation and dogmatic intolerance, revealing that people on the extremes of the political spectrum are more rigid and dogmatic in their world views. (C) A linear relationship between political orientation and authoritarianism was observed, with people from the far right of the political spectrum showing more obedience to authorities and conventions. (D) Dogmatic intolerance and authoritarianism were positively correlated, indicating commonality between these two sub-components of radicalism. See also STAR Methods and Figure S1.

A factor analysis of individual items identified three latent factors (Figure 1A; see STAR Methods for detailed information about the factor loadings and their interpretation), which we labeled “political orientation” (loading on leftward versus rightward political views), “dogmatic intolerance” (loading on questions related to intolerance to opposing political beliefs and rigidity of belief system), and “authoritarianism” (loading on questions related to obedience to authorities, adherence to group conventions, and aggression against deviating behavior). Together, these three factors explained 40% of the variance in questionnaire responses. In what follows, we focus on the dogmatic intolerance and authoritarianism factor scores as summary indices of radicalism [23, 24, 25].

Notably, a clear quadratic relationship was evident between political orientation and dogmatic intolerance (β = 0.37, p < 10^−11^), indicating that both the far left and far right of the political spectrum hold similarly intolerant and rigid beliefs, replicating previous findings [28]. On the other hand, a linear relationship of authoritarianism with political orientation was found (β = 0.38, p < 10^−11^), showing that those on the right of the political spectrum displayed higher levels of authoritarianism, also as reported previously [29]. Finally, dogmatic intolerance and authoritarianism were positively correlated (β = 0.21, p < 0.0001).

**Figure 2:**
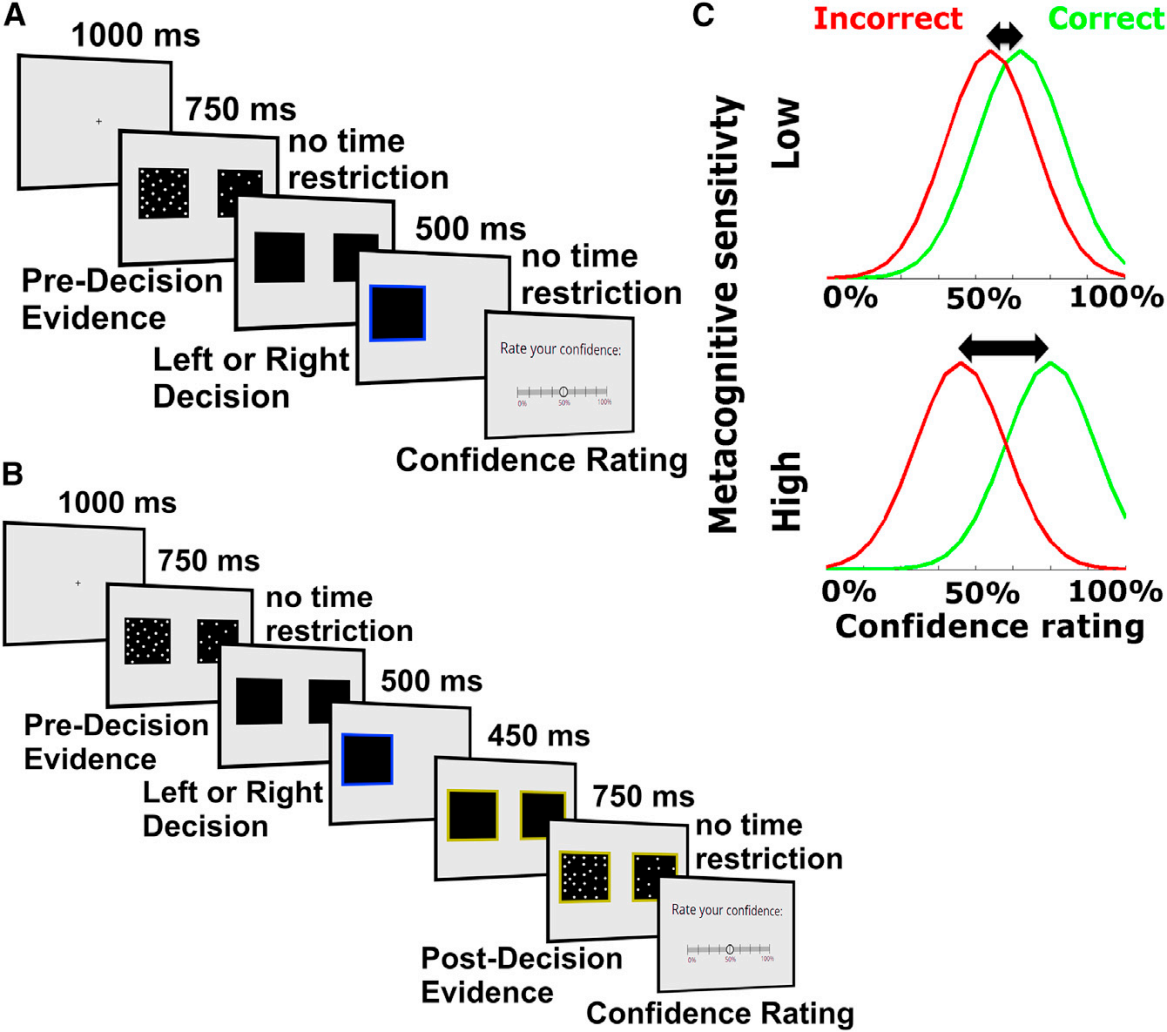
Behavioral Tasks (A) Confidence task (task 1): Participants were asked to judge which of two patches contained a greater number of flickering dots before rating their confidence in each decision. Task difficulty was determined by a fixed difference in dot number between the patches and was individually adjusted in an initial calibration phase to target approximately 71% correct performance. (B) Post-decision evidence integration task (task 2): Participants performed the same perceptual decision as in part (A), but after each decision, they were presented again with a new sample of flickering dots before rating their confidence. In half of trials, participants received the same evidence strength post-decision as pre-decision, while in the other half of trials, they received stronger post-decision evidence (pre-adjusted to a strength that led to 80% performance). (C) Metacognitive sensitivity is defined as the correspondence between task performance and confidence ratings—the extent to which participants rate higher confidence when correct and lower confidence when incorrect. Each graph shows a hypothetical probability distribution over confidence ratings for correct and incorrect trials, with the overlap between distributions determining metacognitive sensitivity. A small separation between these distributions indicates low metacognitive sensitivity (upper), while a large separation indicates high metacognitive sensitivity (lower). See also STAR Methods.

We next investigated whether metacognitive aspects of decision-making predict facets of radicalism. Subjects were asked to carry out a series of perceptual discrimination tasks assaying decision-making and metacognition (Figures 2A and 2B) before filling out the same questionnaires administered in study 1. A first experiment was conducted on a new sample of 381 US participants (study 2), and all key findings were replicated in an independent sample of 417 US participants (study 3). Importantly, we also replicated both the three-factor structure of questionnaire responses observed in study 1 and the pattern of interrelations between factors (quadratic relationship between dogmatic intolerance and political orientation, study 2: β = 0.42, p < 10^−16^; study 3: β = 0.40, p < 10^−15^; linear relationship between authoritarianism and political orientation, study 2: β = 0.32, p < 10^−8^; study 3: β = 0.38, p < 10^−12^; positive association between authoritarianism and dogmatic intolerance, study 2: β = 0.22, p < 10^−4^; study 3: β = 0.29, p < 10^−7^).

In the confidence task (task 1), participants first completed a series of perceptual discrimination judgments as to which of two flickering patches contained a greater density of dots, followed by confidence ratings in their choices. Participants were rewarded according to the extent to which confidence ratings tracked their objective performance over 60 trials and were thus incentivized to report their confidence as accurately as possible. Our measure of interest was metacognitive sensitivity (meta-*d′*) [30], which quantifies subjects’ ability to discriminate correct from incorrect decisions (see Figure 2C for the intuition underpinning this measure). Metacognitive sensitivity is conceptually and empirically distinct from a bias toward reporting higher or lower confidence [7].

**Figure 3:**
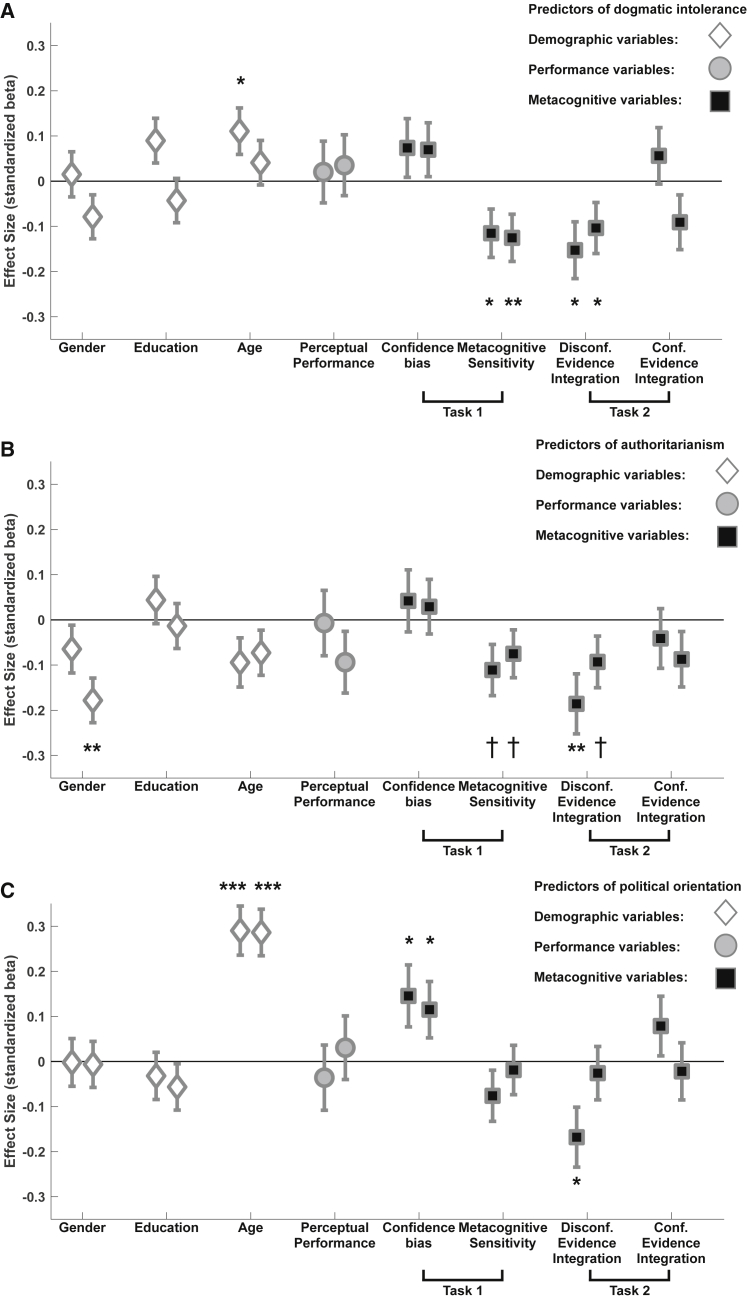
Impaired Metacognitive Sensitivity and Reduced Disconfirmatory Evidence Integration Predict Facets of Radicalism (A–C) Multiple regression analyses predicting factor scores (dogmatic intolerance, authoritarianism, and political orientation) from metacognitive sensitivity and post-decision evidence integration, controlling for multiple demographic variables (gender, education, age) and other task-related variables (e.g., performance in the perceptual decision task). Perceptual performance was averaged across tasks 1 and 2. We present standardized beta coefficients ± SE of predictors for study 2 (left markers, n = 381) and study 3 (right markers, n = 417). (A) Dogmatic intolerance was associated with impaired metacognitive sensitivity and reduced disconfirmatory evidence integration, in the absence of differences in overconfidence or performance. (B) Authoritarianism showed qualitatively similar patterns of association as dogmatism. (C) Political orientation (higher values represent more conservative views) was consistently associated with a bias toward overconfidence but not changes in metacognitive sensitivity or post-decision evidence integration. Effects in study 3 were tested one-tailed based on the directional hypothesis derived from study 2. ^*†*^p < 0.1, ^∗^p < 0.05, ^∗∗^p < 0.01, ^∗∗∗^p < 0.001. Task 1, confidence task; task 2, post-decision evidence integration task. See also Figures S3 and S4.

**Figure 4:**
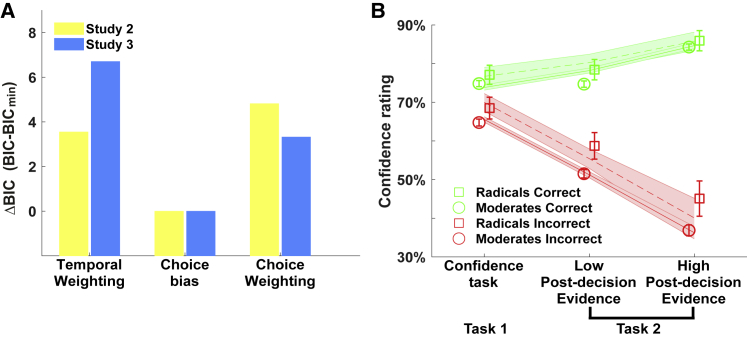
Individual Differences in Radicalism Are Captured by a Choice Bias Model (A) A choice bias model fitted to the confidence data across both tasks best accounted for variations in a composite measure of radicalism (summed factor scores of dogmatic intolerance and authoritarianism). We compared among three computational models within multiple regressions that predicted radicalism from fitted model parameters. We present the BIC of each regression against the lowest BIC in the model set (the best model has a difference in BIC of zero). (B) Radicals reduce their confidence less when new evidence indicates they are wrong (reduced disconfirmatory evidence integration). To visualize this effect, we combined data from study 2 and study 3 and compared the 10% most radical participants (based on the composite measure) against the rest of the sample. Aggregate confidence ratings are separated according to whether the decision was correct (green) or incorrect (red). Markers (circles and squares) show raw data (group averages ±95% confidence interval) for each condition. Lines (solid line, moderates; dashed line, radicals) show posterior predictives from the choice bias model. Predictions were simulated from best-fitting parameters and represent group averages ±95% confidence interval. Task 1, confidence task; task 2, post-decision evidence integration task. See also STAR Methods and Figures S3 and S4.

In line with our hypothesis, higher values of dogmatic intolerance were associated with reduced metacognitive sensitivity (study 2: β = −0.12, p = 0.032, R^2^ = 0.01; see Figure 3A), in the absence of any effect on perceptual discrimination performance (study 2: β = 0.02, p = 0.77) and controlling for key demographic variables (i.e., age, gender, education). Importantly, there was also no relation between dogmatism and overconfidence (study 2: β = 0.07, p = 0.26), suggesting a specific reduction in the sensitivity with which confidence tracks performance, rather than a bias in confidence. We replicated this reduction of metacognitive sensitivity in dogmatic individuals in study 3 (β = −0.13, one-tailed p = 0.008, R^2^ = 0.014), again in the absence of any observed link with perceptual performance (β = 0.04, p = 0.60) or confidence bias (β = 0.07, p = 0.24). These results show that more dogmatic people manifest a lowered capacity to discriminate between their correct and incorrect decisions, after controlling for differences in both primary task performance and confidence bias. We obtained a qualitatively similar pattern for authoritarianism (see Figure 3B), with trends of reduced metacognitive sensitivity (study 2: β = 0.11, p = 0.051; study 3: β = −0.08, one-tailed p = 0.08), but no relation with perceptual performance or confidence bias (all p values > 0.17). Across both facets of radicalism, this failure in metacognition was driven by radicals holding unreasonably high confidence in incorrect decisions compared to moderates (Figures 4B and S4).

In light of long-standing debates about whether the cognitive profile of radicals is more similar to those on the left or right sides of the political spectrum [5, 31], we also tested the relation between political orientation (rightward versus leftward) and metacognition. Here, the pattern of results was qualitatively different, with no reduction of metacognitive sensitivity (study 2: β = −0.08, p = 0.18; study 3: β = −0.02, p = 0.73) in more conservative participants. In contrast, more conservative participants showed an increased bias toward overconfidence (study 2: β = 0.15, p = 0.035, R^2^ = 0.01; study 3: β = 0.12, one-tailed p = 0.033, R^2^ = 0.008; see Figure 3C), as found previously [32].

Metacognitive sensitivity is thought to be strongly linked to an integration of evidence following a decision, allowing latitude for the recognition and reversal of incorrect choices [10, 11, 14]. Having demonstrated a specific decrease in metacognitive sensitivity in more radical participants, we next considered the same participants’ sensitivity to new evidence. To specifically probe such post-decisional processing, in a second phase of the experiment, we inserted an additional sample of evidence (a new series of flickering dots) after subjects had committed to a choice but prior to providing a confidence rating (task 2). Following correct choices, additional evidence should normatively increase participants’ confidence (due to integration of confirmatory evidence; green markers in Figure 4B), whereas for incorrect choices, additional evidence should lead to a decrease in confidence (due to integration of disconfirmatory evidence; red markers in Figure 4B).

In line with the proposal of a post-decisional process supporting metacognition [10], metacognitive sensitivity measured in task 1 explained participants’ sensitivity to post-decision evidence in task 2 (study 2: β = 0.1, p = 0.034; study 3: β = 0.18, one-tailed p < 0.0001). Furthermore, and consistent with a tripartite relationship between radicalism, metacognitive sensitivity and post-decision evidence integration, dogmatic intolerance was associated with a specific reduction in disconfirmatory evidence integration (study 2: β = −0.15, p = 0.016, R^2^ = 0.015; study 3: β = −0.1, one-tailed p = 0.034, R^2^ = 0.008; see Figure 3A), representing a smaller decrease in confidence on incorrect trials. Conversely, there was no association between confirmatory evidence integration on correct trials and dogmatism (study 2: β = 0.06, p = 0.37; study 3: β = −0.09, p = 0.13); i.e., more dogmatic people showed similar increases of confidence on correct trials as that seen in moderates. We again found a similar pattern of results in relation to authoritarianism (see Figure 3B) with decreased disconfirmatory evidence integration in study 2 (β = −0.19, p = 0.005, R^2^ = 0.019) and the same trend in study 3 (β = −0.09, one-tailed p = 0.05, R^2^ = 0.01), despite no effect on confirmatory evidence integration (study 2: β = −0.04, p = 0.53; study 3: β = −0.09, p = 0.16). In contrast, while higher conservatism was related to reduced disconfirmatory evidence integration in study 2 (β = −0.17, p = 0.012), this effect was not replicated in study 3 (β = −0.02, one-tailed p = 0.33).

In light of associations among dogmatic intolerance, authoritarianism, and multiple behavioral measures of metacognitive sensitivity, we next asked whether we could identify a core computational driver of radicalism. We first combined the factor scores of dogmatic intolerance and authoritarianism to construct a composite measure of radicalism. As expected, this combined measure showed similar relationships with metacognition as the individual components (see Figure S3), with impaired metacognitive sensitivity (study 2: β = −0.13, p = 0.0098, R^2^ = 0.018; study 3: β = −0.13, one-tailed p = 0.006, R^2^ = 0.015) and reduced disconfirmatory evidence integration (study 2: β = −0.21, p = 0.001, R^2^ = 0.027; study 3: β = −0.12, one-tailed p = 0.015, R^2^ = 0.011). We next used this score to identify putative mechanisms underpinning reduced metacognitive sensitivity and disconfirmatory evidence integration in more radical participants.

To this end, we compared alternative computational models of how post-decision evidence affects confidence [11, 33] (see STAR Methods for detailed descriptions of the models). All models were grounded in signal detection theory, with two free parameters (*μ*_*low*_ and *μ*_*high*_) representing internal evidence strength for the weak and strong evidence conditions, respectively. The models differed in how they updated their confidence in light of new evidence. A “temporal weighting” model allows an asymmetry in the overall weighting of pre- and post-decision evidence; a “choice bias” model adds evidence for the chosen response, without altering post-decision evidence integration; and a “choice weighting” model incorporates asymmetric weighting of confirmatory and disconfirmatory evidence. We fit the model simultaneously to data from the confidence task (task 1, no post-decision evidence) and the post-decision evidence task (task 2) and compared models based on how well variability in fitted parameters captured individual differences in radicalism in a linear regression.

The “choice bias” model best explained variations in radicalism (difference in Bayesian information criterion [BIC] relative to next best model: study 2 = 3.5 and study 3 = 3.3; see Figure 4A) via a positive association with choice-dependent biases in confidence (study 2: β = 0.14, p = 0.012; study 3: β = 0.18, one-tailed p = 0.0005). This model accounts for a reduction in post-decisional processing in more radical participants by boosting confidence in chosen options, thereby making changes of mind less likely (Figure 4B).

Taken together, our data show that key facets of radicalism are associated with specific alterations in metacognitive abilities. The finding that decision performance per se was not associated with radicalism reveals that a specific change in information processing is manifest at a metacognitive, rather than cognitive, level. Importantly, our results show that radicalism is associated with reductions in metacognitive sensitivity, i.e., the reliability with which subjects distinguish between their correct and incorrect beliefs. Thus, our findings complement and extend previous studies documenting alterations in confidence in political radicals [3, 4, 6] but suggest that these alterations may stem from changes in metacognitive sensitivity. In contrast, for more right-wing subjects (as indexed by political orientation), a change in confidence bias was observed. Without the application of psychophysical measures of metacognition, it has not, up until now, been possible to disentangle these two factors.

What is striking is our demonstration that these impairments are evident during performance of a low-level perceptual discrimination task, where participants are unlikely to have strong *a priori* vested interest in the outcome of their decisions, ruling out multiple possible confounds (e.g., prior knowledge and motivational factors). This contrasts with previous studies that have investigated changes of mind about political attitudes themselves, a context where there exists a strong motivation for people to maintain their current beliefs in order to sustain a positive (and consistent) self-image [15, 16, 17, 18]. Thus, our results suggest a potential explanation for why it is notoriously difficult to change extreme beliefs by what would appear to be the simple expediency of confronting people with evidence that contradicts these beliefs. Before such information can update attitudes, the manner in which a recipient processes this information may need to be altered. We stress, however, that our results are entirely compatible with a complementary role of motivational factors as contributing to the maintenance of radical beliefs, and it is possible that motivational factors may themselves interact with metacognitive abilities.

Our modeling results suggest that a reduction in changes of mind in radicals is driven by a boosting effect of choice, leading participants to assign undue probability to the option they chose without affecting the integration of post-decision evidence. This computational mechanism shares notable similarities with classical findings in psychology in which the act of making a choice itself affects subsequent preferences [34]. In contrast, recent laboratory studies of post-decision evidence integration have found that subjects’ behavior was best described either by a near-optimal Bayesian model [11] or by diminished sensitivity to post-decision evidence [33]. However, since both of these studies investigated small samples of participants, variability in radicalism of political and other beliefs was presumably limited, where, for example, a majority of “moderates” would obviate the need for a choice bias term. We stress that our modeling approach aimed to find a model that best accounts for individual differences in radicalism (while also fitting the overall behavioral pattern). How to reconcile such individual differences with a general model of post-decision evidence integration across different tasks remains a rich topic for future investigation.

In our study, we investigated independent judgments wherein participants integrate two consecutive samples of information. This is distinct from more elaborate beliefs formed over longer timescales, which require integration of multiple samples of information. A useful future extension of our work will be to extrapolate our findings to situations where learning is required over extended periods of time [35, 36]. Our computational model fits indicate that more radical participants assign undue probability to chosen options when updating their confidence, which over repeated exposure to multiple samples of evidence may summate, such that even small asymmetries in information processing could lead to a highly skewed representation of reality. In the current task, such resistance to updating is detrimental, leading to a loss of earnings (Figure S3). However, in other scenarios, such as if there were reason to distrust the fidelity of the new information, a reduction in belief flexibility may prove adaptive. Such considerations remain to be explored in future studies and point to the intriguing notion that metacognitive flexibility may itself be amenable to strategic or environmental influences.

We used perceptual decision-making as a model system that permitted precise control over performance so as to reveal relationships between radicalism and metacognition. A question remains as to whether the metacognitive alterations shown here would extend to other types of decision (e.g., value-based, memory-based). Recent evidence points toward a core domain-general circuit supporting metacognitive abilities [37, 38], suggesting that metacognition as measured in the current task may represent an indicator of a more general metacognitive ability. Despite relatively small effect sizes, our findings linking radicalism to changes in metacognition are robust and replicable across two independent samples. However, we note that other, domain-specific facets of metacognition (e.g., insight into the validity of higher-level reasoning or certainty about value-based choices [39]) are arguably closer to the drivers of radicalization of political and religious beliefs, suggesting that the current results represent a lower bound for the strength of a relationship between metacognitive abilities and radicalism. Similarly, while our measures of radicalism were derived from questionnaires tapping into political attitudes, it is possible that impairments in metacognition may constitute a general feature of radicalism about political, religious, and scientific issues.

## STAR★Methods

### Key Resources Table

**Table:** 

REAGENT or RESOURCE	SOURCE	IDENTIFIER
**Software and Algorithms**		

MATLAB	Mathworks	Matlab_R2017b
R	Bell Laboratories	R 3.3.2
R-STAN	http://mc-stan.org/users/interfaces/rstan	Rstan 2.17.3
jsPsych	https://www.jspsych.org/	JsPsych 5.0.3
Gorilla Experiment Builder	https://gorilla.sc/	
R *psych* package	https://cran.r-project.org/web/packages/psych/index.html	Version 1.8.4
R *nFactors* package	https://cran.r-project.org/web/packages/nFactors/index.html	Version 2.3.3
Custom code (data analysis, computational models)	This paper	https://github.com/metacoglab/RollwageDolanFleming

### Contact for Reagent and Resource Sharing

Further information and requests for resources and reagents should be directed to and will be fulfilled by the Lead Contact, Stephen M. Fleming (stephen.fleming@ucl.ac.uk).

### Experimental Model and Subject Details

All three studies were conducted online and recruited subjects from the US via the online labor market Amazon Mechanical Turk [40]. Mechanical Turk has been shown to be more representative of the population than typical college student samples [41, 42], and produces high quality data [40] with good internal and external validity [43, 44], even when using complex behavioral tasks [45]. All data were collected in the year 2017. Subjects gave informed consent and the study was approved by the Research Ethics Committee of University College London (#1260-003).

In Study 1 subjects completed questionnaires about political issues [4, 19, 20, 21, 22]. In Studies 2 and 3, participants filled out the same questionnaires as in Study 1 and additionally completed two perceptual decision-making tasks (a confidence task and a post-decision evidence integration task; see below). In Study 3, we additionally included the Zung self-rating depression scale and a question about self-esteem; these data will be reported elsewhere.

In Study 1 we analyzed data from 344 subjects (46% women, mean age 34.9 years, range 19-73 years; see Figure S1A left panel). In Study 2 a sample of 381 subjects were included for analysis of questionnaires and behavioral tasks (51.4% women, mean age 36.0 years, range 19-70 years; see Figure S1A middle panel). In Study 3 a sample of 417 subjects was included for analysis of questionnaires and behavioral tasks (47% women, mean age 35.8 years, range 18-71 years; see Figure S1A right panel). The sample size of Study 3 (n = 417) was defined by an *a priori* power analysis based on effect sizes from Study 2 for associations between radicalism and meta-*d’* (power = 77%) or disconfirmatory evidence integration (power = 89%). All studies recruited participants with a diverse education level (see Figure S1B), which is generally comparable to the US population [46].

Self-reported political orientation (“very liberal” = 0 to “very conservative” = 100) in both samples was somewhat skewed to the liberal end of the spectrum (Study 1: mean = 38.1; Study 2: mean = 38.0; Study 3: mean = 41.9) which is in line with previous reports of Amazon Mechanical Turk workers being more liberal than the general US population [41]. However, there remained substantial variability in political orientation as measured via factor analysis.

### Method Details

#### Experimental design

##### Stimuli

Perceptual discrimination experiments were programmed in JavaScript using JsPsych (version 5.0.3) and hosted on the online research platform Gorilla (https://gorilla.sc/). The experiment was accessed via a web browser and participants were required to use full-screen mode to complete the task. Stimuli consisted of two black squares (each 250 × 250 pixels) centrally positioned on the screen, one square to the left and the other square to the right of center. These squares were subdivided into grids of 625 cells, randomly filled with white dots. One square always contained 313 cells filled with dots and the other square contained a greater number of filled cells (the exact number of additional dots was adjusted for each individual using a staircase procedure). The difference in dot number between the two squares determined the judgment difficulty. Five such configurations were presented per trial, each for 150ms, creating the impression of flickering dots. The exact location of dots per configuration was random, but within one trial, the difference in number of dots and also the side which contained more dots remained constant.

##### Task and procedure

For Studies 2 and 3, the experiment lasted around 1 hour. After receiving general information and instructions, participants began the behavioral experiment which was divided into 3 parts. First, participants completed 120 trials of a calibration phase (which was used to individually adjust the task difficulty, see below), in which they performed the perceptual judgement by reporting whether the left or the right square contained more dots. Second, there were 60 trials of the same perceptual judgement followed by a confidence rating (“confidence task,” Task 1; Figure 2A). Finally, there were 120 trials of a “post-decision evidence integration task” (Task 2; Figure 2B), in which subjects performed the perceptual judgement, received additional post-decision evidence and then rated their confidence. After completing the behavioral tasks, participants filled out questionnaires regarding their political orientation and radicalism.

##### Calibration phase

Before performing the main task, each participant performed a calibration phase comprising 120 trials judging whether the left or the right square contained more dots without confidence ratings, using their computer keyboard (using the “W” and “E” keys to indicate left and right, respectively). Responses were unspeeded and possible only after stimulus offset. During the calibration phase (but not the experimental phase), visual feedback was delivered to indicate whether the judgment was correct (a green frame around the chosen square) or incorrect (a red frame around the chosen square). The calibration phase was used to find a stimulus strength (dot difference between left and right) for each participant that elicited approximately 71% correct performance (actual performance: *mean* = 73.2%, *sd* = 6.3%) in the discrimination task using a 2-down-1-up staircase procedure [47] operating on the logarithm of dot difference. Participants completed 70 trials of the staircase and the average of the last 25 trials was stored and used as the individual stimulus strength throughout the rest of the experiment. For the post-decision evidence integration task we also presented stronger evidence than the staircased stimulus strength on a subset of trials. This stronger level of evidence was generated by multiplying the logarithm of the staircased dot difference by a factor of 1.3. To quantify the performance level induced by this stimulus strength for each individual, participants performed 50 additional perceptual judgements at this higher stimulus strength interleaved with the staircased trials (after 20 initial “burn-in” trials to allow the staircase to converge) and yoked to the current staircase value. In the group as a whole, the higher evidence strength evoked a mean performance level of 81.0% correct (*sd* = 3.8%).

##### Confidence task (Task 1)

The confidence task comprised a total of 60 trials, all at the same (lower) stimulus strength determined in the calibration phase. On each trial, participants judged which side contained more dots, before rating confidence in their decision (a detailed trial timeline is displayed in Figure 2A). Participants were instructed to report their confidence as a subjective probability that their decision was correct, rated on a 9-point sliding scale. The scale midpoint was labeled with 50%, the lowest category with 0% and the highest category with 100%. Confidence ratings were incentivized using the quadratic scoring rule [48]. This scoring rule provides maximal reward both when one is maximally confident and right, and minimally confident and wrong.

##### Post-decision evidence integration task (Task 2)

The post-decision evidence integration task consisted of 120 trials, split into 60 trials with low post-decision evidence strength and 60 trials with high post-decision evidence strength, pseudo-randomly interleaved. Within each trial, participants first judged which side contained more dots as described under “Confidence task” above. After this initial decision, they received additional evidence. The location of higher dot density in the post-decision evidence presentation was always of the same (correct) sign as the pre-decision evidence presentation, but of variable strength. Subjects were instructed that this evidence was “bonus” information that could be used to inform their confidence in their initial response. The post-decision evidence could either have the same strength as the pre-decision evidence (low post-decision evidence) or have a higher evidence strength (high post-decision evidence). After the presentation of post-decision evidence, participants were asked to indicate their confidence in their initial decision.

#### Data quality and exclusion criteria

In Study 1 we analyzed questionnaire data from 344 subjects. Six subjects were excluded from the original sample (n = 350) because they failed to answer correctly at least one of two catch questions interspersed within the questionnaires (“If you have read this question please choose *Agree Completely*” and “Please choose *Disagree completely* if you read this question”).

In Study 2 a sample of 381 subjects were included for analysis of questionnaires and behavioral tasks. To ensure data quality we excluded 123 subjects (original sample n = 504) based on a range of pre-defined exclusion criteria. First, 5 subjects were excluded from questionnaire analysis due to answering at least one of the two catch questions incorrectly, as described above. An additional 77 subjects were excluded due to performance in the perceptual decision-task being above 85% or below 60% correct, indicating that the staircase procedure was insufficient to produce threshold performance. Five subjects were excluded because they chose a single confidence rating more than 90% of the time and an additional 10 subjects were excluded due to median confidence response times of below 850 ms, indicating that they rated their confidence very quickly and possibly without care (see Figure S2A left panel). Finally, 26 subjects were excluded due to a large proportion of missed trials (> 5%).

In Study 3 a sample of 417 subjects was included for analysis of questionnaires and behavioral tasks. As in Study 2, we excluded 158 subjects (original sample n = 575) based on the same pre-defined exclusion criteria. Seventeen subjects were excluded from questionnaire analysis due to answering at least one of the two catch questions incorrectly. An additional 90 subjects were excluded due to performance in the perceptual decision task being above 85% or below 60%, indicating that the staircase procedure was insufficient to produce threshold performance. 11 subjects were excluded because they chose a single confidence rating more than 90% of the time, and an additional 19 subjects were excluded for median confidence reaction times below 850 ms (see Figure S2A right panel). Finally, 21 subjects were excluded due to a significant proportion of missed trials (> 5%).

Exclusion criteria followed similar procedures used in our lab [9] and elsewhere [49]. The overall exclusion rate (∼25%) was similar to other studies from our lab and is consistent with a recent meta-analysis which found that between 3% and 37% of the sample is typically excluded in web-based experiments [50].

We applied these exclusion criteria to ensure high-quality data and prevent our results being influenced by people performing the task without care. However, we also established that results were qualitatively similar in the absence of exclusions, with our composite measure of radicalism remaining associated with impaired metacognitive sensitivity (Study 2: β = −0.13, p = 0.008; Study 3: β = −0.12, p = 0.01) and reduced disconfirmatory evidence integration (Study 2: β = −0.22, p = 0.0002; Study 3: β = −0.14, p = 0.009).

#### Behavioral analysis

##### Measurement of metacognitive ability

For assessment of metacognitive ability we calculated meta-*d’* [30], a signal detection theoretic measure of metacognitive sensitivity that is uncorrupted by the tendency to report high or low confidence (overconfidence bias [7]). To estimate *meta-d’* we employed a Bayesian estimation scheme [51] (HMeta-d; https://github.com/metacoglab/HMeta-d), using the non-hierarchical version of the model.

##### Measurement of post-decision evidence integration

We measured confirmatory and disconfirmatory evidence integration as changes in confidence induced by post-decision evidence. We constructed trial-by-trial linear models for every participant, separately for correct and incorrect trials across data pooled across Tasks 1 and 2, using post-decision evidence strength as a predictor (confidence task = 0, low post-decision evidence = 1, high post-decision evidence = 2) and confidence ratings as the dependent variable. Individual beta weights for correct trials, indicating increases of confidence due to post-decision evidence, were estimated as measures of confirmatory evidence integration. Disconfirmatory evidence integration was estimated as the beta weight on incorrect trials (we reversed the sign of this beta weight in the figures such that higher values indicate greater disconfirmatory evidence integration).

We additionally tested whether sensitivity to post-decision evidence could be predicted from metacognitive sensitivity measured in Task 1. For this purpose, we calculated sensitivity to post-decision evidence based solely on trials from Task 2 to ensure independence from estimation of metacognitive ability in Task 1. For each subject we constructed a trial-by-trial linear model with confidence as dependent variable, entering the following predictors: accuracy (correct = 1 and incorrect = −1), post-decision evidence strength (low post-decision evidence = 1 and high post-decision evidence = 2) and the critical accuracy × post-decision evidence strength interaction. This interaction term quantifies the extent to which confidence increases on correct trials and decreases on error trials at higher levels of post-decision evidence strength, thus forming a summary measure of sensitivity to additional evidence.

#### Factor analysis

There is extant debate about the underlying structure of political ideology and its relation to radical beliefs [52]. Therefore, instead of relying on direct self-report measures of political orientation and radicalism, we combined multiple questionnaires related to political orientation, intolerance, dogmatism and authoritarianism, and conducted a factor analysis to identify the most parsimonious factor structure.

Regarding political orientation, we included questions that reflect putatively separate facets of social and economic conservatism [52]. Participants filled out questions about the following issues: political orientation on a “liberal-conservative” dimension (general conservatism and separately for social and economic issues), voting behavior and identification with the U.S. Democratic or Republican party, a social and economic conservatism scale [19], and attitudes toward specific political issues [4].

To measure dogmatism we employed a widely used scale that assays this construct [22]. Additionally, we administered previously used questions [4] about belief superiority and intolerance of opposing political opinions as these are known to show considerable conceptual overlap with dogmatism and have previously been reported as manifesting a quadratic relationship with political orientation [4].

Authoritarianism is widely conceptualized as prevalent on the right side of the political spectrum together with more controversial proposals of a similar trait in left-wing individuals [5, 21]. Left-wing authoritarianism may be rarely reported due to problems with measurement (right-wing authoritarianism scales are not content-free but target conservative tendencies) or sample characteristics. To counteract such concerns here we included both left-wing [21] and right-wing [20] authoritarianism scales.

An exploratory factor analysis was conducted on all 78 single questionnaire items using maximum likelihood estimation. Factor analysis was conducted using the fa() function from the Psych package in R, with an oblique rotation (oblimin). The number of factors was extracted based on the Cattell-Nelson-Gorsuch [53] test using the nFactors package in R. The Cattell-Nelson-Gorsuch test revealed a three-factor solution as the best and most parsimonious solution for the covariance structure of the single items (see Figure 1A and Figure S1C for the factor loadings of individual items). The pattern of factor loadings was qualitatively similar for both Study 1 (Figure 1A) and the combined data from all three Studies (Figure S2C). To obtain precise estimates of factor loadings and thus more reliable factor scores we conducted the factor analysis on the pooled sample from all three studies when extracting factor scores for use in analysis of behavioral data in Studies 2 and 3.

The first factor tracked political orientation (liberal to conservative) as indicated by the highest loading item *Please rate your overall political attitude on the dimension from “liberal” to “conservative”* (factor loading = 0.90). The second factor loaded predominantly on the dogmatism and intolerance questionnaires, with the highest loading items concerning rigid and dogmatic world views, e.g., *My opinions are right and will stand the test of time* (factor loading = 0.73) and intolerance of opposing political beliefs, e.g., *My beliefs about the government’s role in helping people in need are totally correct (mine is the only correct view)* (factor loading = 0.58). The third factor was related to authoritarianism and showed the highest loadings on questions related to obedience to in-group authorities, e.g., *A revolutionary movement is justified in demanding obedience and conformity of its members* (factor loading = 0.43), group conventions, e.g., *The withdrawal from tradition will turn out to be a fatal fault one day* (factor loading = 0.37) and support of aggression to reach one’s political goals, e.g., *What our country really needs is a strong, determined Chancellor which will crush the evil and set us on our right way again* (factor loading = 0.40). Although we labeled these three factors based on both theoretical considerations and their respective patterns of item loadings, we acknowledge that this is an inherently subjective process and that alternative labels are possible. However we stress that the identification of the factors, and their interrelationships, was entirely data driven and was replicated across all three experiments. We note that this pattern of factor loadings is consistent with a one-dimensional model of political orientation (in contrast to separate factors for social and economic conservatism).

#### Computational modeling

All computational models were adapted from those developed by Fleming et al. [11] in a study of post-decision evidence integration during random-dot kinematogram decisions. We examined the potential of these models to explain individual differences in metacognitive sensitivity and confidence updating based on post-decision evidence observed in relation to radicalism. All models were grounded in signal detection theory, and simultaneously modeled choices and confidence ratings of Tasks 1 and 2. In the confidence task, subjects receive one internal sample, *X*_*pre*_ generated from pre-decision evidence, whereas for the post-decision evidence integration task subjects receive two internal samples, *X*_*pre*_ from pre-decision evidence and *X*_*post*_ from post-decision evidence. These samples in turn were generated from a Gaussian whose sign depended on the location of objectively higher dot density (left = −1, right = 1) and mean on internal evidence strength *θ*_pre_ or *θ*_post_:

$$X_{pre}∼N\left(d{\text{θ}}_{pre},1\right)$$

$$X_{post}∼N\left(d{\text{θ}}_{post},1\right)$$

The internal evidence strength depended on the dot difference and was always the same for *θ*_pre_ (*μ*_*low*_), whereas the evidence strength could be either low or high for *θ*_post_ ∈ [*μ*_*low*_ *μ*_*lhigh*_], where *μ*_*low*_ and *μ*_*high*_ are free parameters. The likelihood of *X*_*pre*_ or *X*_*post*_ was approximated by a Gaussian with mean μ and variance σ^2^. For the confidence task (Task 1) in which only *X*_*pre*_ was presented, we set μ = *θ*_pre_ and σ^2^ = 1. For the post-decision evidence task (Task 2), we approximated the likelihood of both *X*_*pre*_ and *X*_*post*_ as a single Gaussian with mean μ and variance σ^2^ determined by a mixture of Gaussians across the two possible evidence strengths. Starting with *X*_*post*_ :

$$P\left(X_{post}|d=1\right)=\sum_{{\text{θ}}_{\text{post}}}p\left({\text{θ}}_{\text{post}}\right)N\left({\text{θ}}_{\text{post}},1\right)$$

As each of the two evidence strengths is equally likely by design $\left(p\left({\text{θ}}_{\text{post}}\right)=0.5\right)$ we can define the mean as:

$$\mu =\frac{\sum \theta_{post}}{2}$$

The aggregate variance σ^2^ can be decomposed into both between- and within condition variance. From the law of total variance:

$${\text{σ}}^{2}=\sum_{{\text{θ}}_{\text{post}}}p\left({\text{θ}}_{\text{post}}\right){\left[E\left[X_{post}|{\text{θ}}_{\text{post}}\right]-\text{μ}\right]}^{2}+\sum_{{\text{θ}}_{\text{post}}}p\left({\text{θ}}_{\text{post}}\right)Var\left(X_{post}|{\text{θ}}_{\text{post}}\right)$$

$${\text{σ}}^{2}=\sum_{{\text{θ}}_{\text{post}}}p\left({\text{θ}}_{\text{post}}\right){\left[E\left[X_{post}|{\text{θ}}_{\text{post}}\right]-\text{μ}\right]}^{2}+1$$

We assume that subjects are agnostic about the set of evidence strengths presented before and after the decision, such that *μ* and σ^2^ are the same for both *X*_*pre*_ and *X*_*post*_.

In both tasks, actions *a* are made by comparing *X*_*pre*_ to a criterion parameter *m* that accommodates any stimulus-independent biases toward the leftward or rightward response, *a* = *X*_*pre*_ > *m*. Each piece of evidence, *X*_*pre*_ and *X*_*post*_, updates the log posterior odds of the rightward location containing a higher dot density, *LO*_*dir*_, which under flat priors is equal to the log likelihood:

$$LO_{dir}^{pre}=log\frac{P\left(d=1|X_{pre}\right)}{P\left(d=-1|X_{pre}\right)}=log\frac{P\left(X_{pre}|d=1\right)}{P\left(X_{pre}|d=-1\right)}$$

$$LO_{dir}^{post}=log\frac{P\left(d=1|X_{post}\right)}{P\left(d=-1|X_{post}\right)}=log\frac{P\left(X_{post}|d=1\right)}{P\left(X_{post}|d=-1\right)}$$

where, due to the Gaussian generative model for *X*, *LO*_*dir*_ is equal to:

$$LO_{dir}=\log\frac{e^{{\left(\text{μ}+X\right)}^{2}/2\sigma^{2}}}{e^{{\left(\text{μ}-X\right)}^{2}/2\sigma^{2}}}$$

Positive values indicate greater belief in the higher dot density being on the right; negative values indicate greater belief in higher density on the left. To update confidence in one’s choice, the belief in dot density (*LO*_*dir*_) is transformed into a belief about decision accuracy (*LO*_correct_) conditional on the chosen action:

If *a* = 1:

$$LO_{correct}=LO_{dir}$$

Otherwise:

$$LO_{correct}=-LO_{dir}$$

As for *LO*_*dir*_, *LO*_*correct*_ in the post-decision evidence task can be decomposed into pre- and post-decisional parts:

$$LO_{correct}^{total}=LO_{correct\;}^{pre}+LO_{correct\;}^{post}$$

For trials of the confidence task, $LO_{correct\;}^{post}$ was set to zero for all models as in those trials no post-decision evidence was presented. The final log odds correct is then transformed to a probability to generate a confidence rating on a 0-1 scale:

$$Confidence=\frac{1}{1+\text{exp}\left(-LO_{correct}^{total}\right)}$$

##### Model extensions accounting for differences in post-decision evidence integration

We considered different mechanisms that could account for reduced metacognitive sensitivity and changes of mind in radicals, adapted from Fleming et. al. [11] and Bronfman et al. [33].

*Temporal weighting*: We considered participants may apply differential weighting to pre- and post-decision evidence when computing confidence. The “temporal weighting” model captured such differences via two free parameters (*w*_pre_ and *w*_post_) as follows:

$$LO_{correct}^{total}=w_{pre}\text{∗}LO_{correct\;}^{pre}+w_{post}\text{∗}LO_{correct\;}^{post}$$

*Choice weighting*: An alternative model applies differential weighting to post-decision evidence depending on whether this evidence is in support of the chosen option (confirmatory) or unchosen option (disconfirmatory). To capture this effect we introduced two separate weighting parameters based on the correspondence between the decision and post-decision evidence:

If sign(*X*_*post*_) = sign(*a*):

$$LO_{correct}^{total}=LO_{correct\;}^{pre}+w_{confirmatory}\text{∗}LO_{correct\;}^{post}$$

Otherwise:

$$LO_{correct}^{total}=LO_{correct\;}^{pre}+w_{disconfirmatory}\text{∗}LO_{correct\;}^{post}$$

*Choice bias*: Finally we considered a model in which subjects become more confident in the option they chose, irrespective of the strength of post-decision evidence. This was implemented by adding a fixed amount of subjective probability to the chosen option which was controlled by a free parameter *w*_*bias*_:

If *a* = 1:

$$LO_{bias}=\text{log}\left(\frac{w_{bias}}{1-w_{bias}}\right)$$

Otherwise:

$$LO_{bias}=\text{log}\left(\frac{1-w_{bias}}{w_{bias}}\right)$$

$$LO_{dir}^{total}=LO_{dir\;}^{pre}+LO_{dir\;}^{post}+LO_{bias}$$

If *a* = 1:

$$LO_{correct}^{total}=LO_{dir}^{total}$$

Otherwise:

$$LO_{correct}^{total}=-LO_{dir}^{total}$$

Note that the choice bias term (unlike the weighting parameters in alternative model extensions) also affects the predictions for the confidence task as it is applied independently of the level of post-decision evidence.

##### Model fitting

We used variational Bayesian inference implemented in STAN [54] to approximate draws from the posterior distribution of parameters given the world state *d*, subjects’ choices *a* and their confidence ratings *r*. Since we had relatively few trials per subject, we used a hierarchical fitting procedure. We set the maximum number of iterations to 150,000 and a convergence tolerance on the relative norm of the objective to 0.0001 (this is a conservative approach regarding convergence; default options in STAN are 10,000 iterations and a convergence tolerance of 0.01). From the approximate posterior, 1000 samples were drawn for each of the following parameters (where ∼indicates “ is distributed as,” *N* represents a normal distribution, *HN* indicates a positive half-normal distribution, and *j* indexes each subject):

Group-level parameters:

$${\text{μ}}_{m}∼N\left(0,1\right)$$

$${\text{σ}}_{m}∼HN\left(0,10\right)$$

$${\text{σ}}_{report}∼HN\left(0,0.1\right)$$

If *Temporal weighting model*:

$${\text{μ}}_{wpre}∼N\left(1,1\right)$$

$${\text{σ}}_{wpre}∼HN\left(0,10\right)$$

$${\text{μ}}_{wpost}∼N\left(1,1\right)$$

$${\text{σ}}_{wpost}∼HN\left(0,10\right)$$

If *Choice weighting model*:

$${\text{μ}}_{wconfirm}∼N\left(1,1\right)$$

$${\text{σ}}_{wconfirm}∼HN\left(0,10\right)$$

$${\text{μ}}_{wdisconfirm}∼N\left(1,1\right)$$

$${\text{σ}}_{wdisconfirm}∼HN\left(0,10\right)$$

If *Choice bias model*:

$${\text{μ}}_{wbias}∼N\left(.5,1\right)$$

$${\text{σ}}_{wbias}∼HN\left(0,10\right)$$

Subject-level parameters:

$${\text{m}}_{j}∼\text{N}\left({\text{μ}}_{m},{\text{σ}}_{m}\right)$$

$${\text{μ}}_{low,j}∼N\left(\frac{d_{low,j}^{\text{'}}}{2},1\right)$$

$${\text{μ}}_{high,j}∼N\left(\frac{d_{high,j}^{\text{'}}}{2},1\right)$$

$$\theta_{pre}={\text{μ}}_{low,j}\;\theta_{post}=\left[{\text{μ}}_{low,j},{\text{μ}}_{high,j}\right]$$

If *Temporal weighting model*:

$$w_{pre,j}∼N\left({\text{μ}}_{wpre},{\text{σ}}_{wpre}\right)$$

$$w_{post,j}∼N\left({\text{μ}}_{wpost},{\text{σ}}_{wpost}\right)$$

If *Choice weighting model*:

$$w_{confirm,j}∼N\left({\text{μ}}_{wconfirm},{\text{σ}}_{wconfirm}\right)$$

$$w_{disconfirm,j}∼N\left({\text{μ}}_{wdisconfirm},{\text{σ}}_{wdisconfirm}\right)$$

If *Choice bias model*:

$$w_{bias,j}∼N\left({\text{μ}}_{wbias},{\text{σ}}_{wbias}\right)$$

Model:

$$X_{pre}∼N\left(d\theta_{pre},1\right)$$

$$X_{post}∼N\left(d\theta_{post},1\right)$$

$$a∼\text{Bernoulli}\_\text{logit}\left(1000\text{∗}\left({\text{X}}_{pre}-m_{j}\right)\right)$$

$$r∼N\left(conf,{\text{σ}}_{report}\right)$$

where “conf” is the output of the confidence computation detailed above. The logit function implements a steep softmax relating *X*_pre_ to *a*, and is applied for computational stability. The mapping between model confidence and observed confidence allowed a small degree of imprecision in the subjects’ ratings via a free parameter *σ*_*report*_ which was fitted at the group level. Note that the internal evidence strengths for the low and high evidence conditions were not fitted hierarchically, but were constrained by subjects’ observed *d’* values.

##### Model comparison

To compare between alternative models we assessed their ability to capture individual differences in radicalism. To this end, we constructed separate multiple regressions to predict radicalism scores from the mean of the posterior draws of each model’s fitted parameters. For each model we inputted *μ*_*low,j*_ and *μ*_*high,j*_ as predictors together with model-specific parameters (choice bias (*w*_bias_), weighting parameters for confirmatory and disconfirmatory evidence (*w*_confirmatory_ and *w*_disconfirmatory_) or weighting parameters for pre and post-decision evidence (*w*_pre_ and *w*_post_)). We computed BIC scores for each multiple regression to identify model fits that best explained individual difference in radicalism. Note that this model comparison approach differs from standard approaches in that it is concerned with best capturing individual differences rather than an aggregate fit to the group.

Since the BIC score includes a penalty for model complexity (i.e., number of parameters), we wished to ensure that the choice bias model was not favored due to its lower complexity alone. We therefore also considered multiple regressions that included only one of the fitted parameters from the more complex temporal weighting and choice weighting models (*w*_disconfirmatory_ or *w*_confirmatory_; *w*_pre_ or *w*_post_) as predictors and included these variants in the model comparison. The parameter combinations with the lowest BIC scores are presented in Figure 4A.

##### Model simulations

To visualize qualitative features of computational model fits and determine their ability to account for the patterns of confidence ratings in moderates and radicals (a posterior predictive check, see Figure 4B), we drew 100 samples from the posterior distributions of fitted parameters for each participant, and for each draw simulated 4000 trials per subject per condition (confidence task, low and high post-decision evidence) with these parameter settings.

### Quantification and Statistical Analysis

In all regression analyses we employed robust fits by using the default robust option of the MATLAB function *fitlm* which applies a “bisquare” weighting. We checked for multicollinearity of all multiple regressions by calculating the variance inflation factor for each predictor, which was < 2 for all regressions and predictors and below a standard cut-off value of 10 [55]. *R*^*2*^-values for each predictor were calculated by comparing the explained variance of the full model including this predictor to a model excluding the predictor of interest. All effects for Studies 1 and 2 were tested two-tailed. Since we had strong *a priori* hypotheses in the replication sample, effects in Study 3 were tested one-tailed based on the directional hypothesis derived from Study 2.

The following regression analyses were conducted:

1. To investigate the relation between political orientation, dogmatic intolerance and authoritarianism, we specified separate models with dogmatic intolerance and authoritarianism as dependent variables and political orientation as a predictor in a second-order polynomial regression, with both linear and quadratic terms for the predictor. The relationship of political orientation with dogmatic intolerance and authoritarianism was labeled as linear or quadratic via comparison of BIC scores of models with either or both terms.
2. To quantify the link between metacognitive sensitivity (measured in the confidence task) and sensitivity to post-decision evidence we conducted a multiple regression analysis with post-decision evidence sensitivity as the dependent variable and separate predictors for meta-*d’* in Task 1, perceptual task performance (*d’*) averaged across Tasks 1 and 2, confidence bias in Task 1, objective evidence strength (logarithm of dot difference) and performance at higher post-decision evidence strength (as measured in the calibration phase).
3. To investigate the relation between metacognitive function and radicalism we implemented separate multiple regression models with the factor scores (dogmatic intolerance, authoritarianism and political orientation) as dependent variables and separate predictors for meta-*d’* in Task 1, confidence bias in Task 1, confirmatory evidence integration in Task 2, disconfirmatory evidence integration in Task 2, perceptual task performance (*d’*) averaged across Tasks 1 and 2, objective stimulus strength (logarithm of dot difference), performance at the higher post-decision evidence strength (as measured in the calibration phase), age, gender and education.
4. Finally, to investigate whether radicalism was associated with reduced earnings in the task, we constructed a multiple regression model with earnings as dependent variable and radicalism as predictor, controlling for perceptual task performance (*d’*) averaged across Tasks 1 and 2 and objective stimulus strength (logarithm of dot difference).

### Data and Software Availability

Fully anonymised data are available from the corresponding author on reasonable request. Code for data analysis and computational model fits are available from a dedicated Github repository (https://github.com/metacoglab/RollwageDolanFleming).

## Supplemental Information

Document S1. Figures S1–S4

Document S2. Article plus Supplemental Information
