# Supplementary material for: Metacognitive Failure as a Feature of Those Holding Radical Beliefs
Source: Curr Biol. 2018 Dec 17;28(24):4014–4021.e8. doi: 10.1016/j.cub.2018.10.053 (PMC6303190; doi:10.1016/j.cub.2018.10.053)
Supplement: Document S1. Figures S1–S4 [file mmc1.pdf]

Current Biology, Volume *28*

Supplemental Information

Metacognitive Failure as a Feature of Those Holding Radical Beliefs

Max Rollwage, Raymond J. Dolan, and Stephen M. Fleming

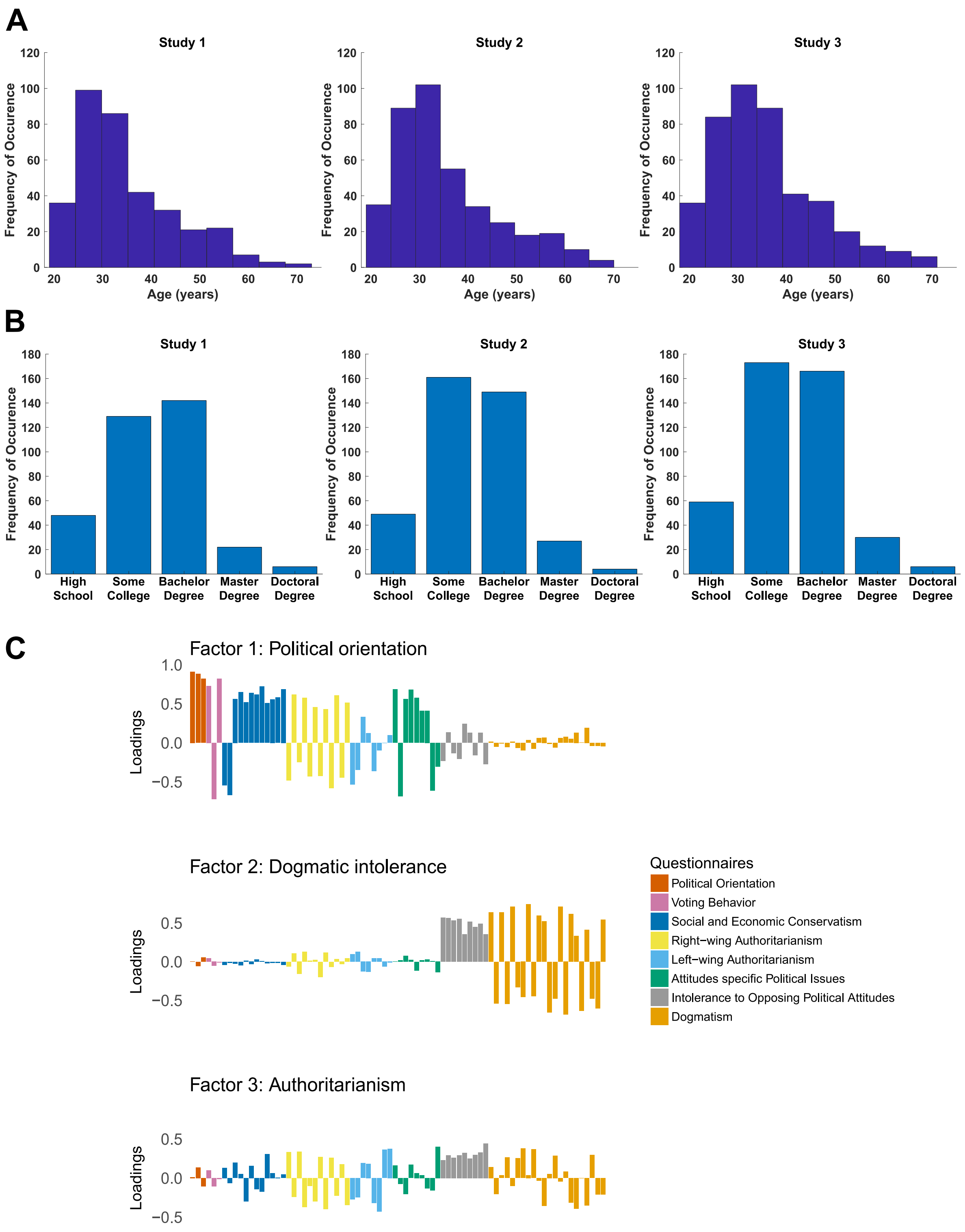

Figure S1. Demographics and factor analytic results for all three studies, related to Figure 1 & STAR methods. A. The bar plots show histograms for the age distributions in Study 1 (N=344, left panel), Study 2 (N=381, middle panel) and Study 3 (N=417, right

panel). **B.** The bar plots show histograms for the education distributions in Study 1 (N=344, left panel), Study 2 (N=381, middle panel) and Study 3 (N=417, right panel). **C.** Factor loadings for the three extracted factors are presented, based on the pooled sample from Study 1, 2 and 3.

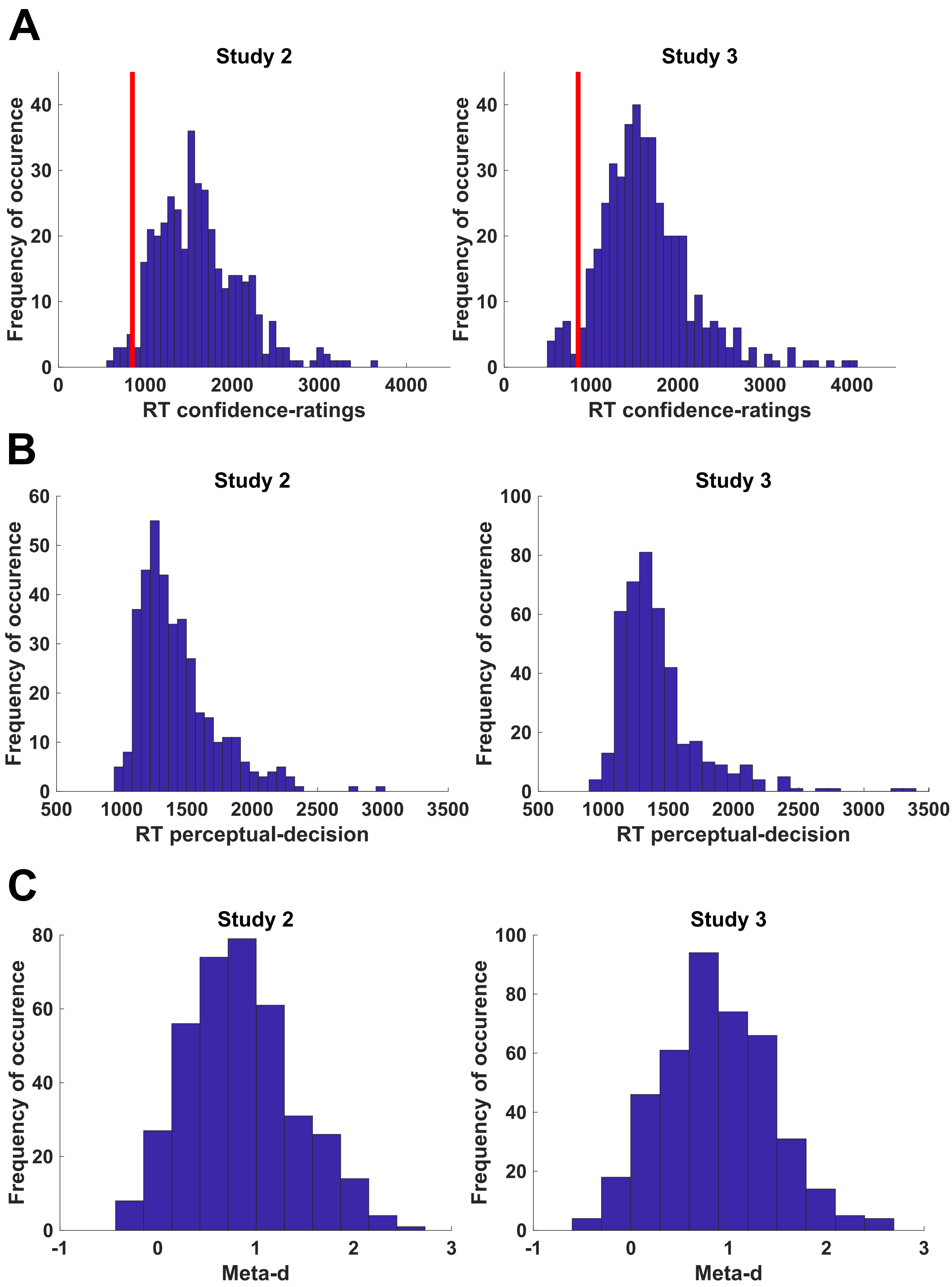

Figure S2. Distributions of meta-d' and median reaction times for perceptual decisions and confidence ratings, related to Figure 2 & STAR methods. A. Median confidence

rating reaction time distribution. Bar plots show histograms of median confidence reaction times in Study 2 (left panel) and Study 3 (right panel) before excluding subjects based on this criterion, but after applying all the other exclusion criteria. The red line indicates the criterion of 850 ms for excluding subjects with median reaction below this criterion. Note that trials with very long reaction times (>15sec) were excluded from all analysis. **B.** Median perceptual decision reaction time distribution. Bar plots show histograms of median reaction times to the perceptual decision after the application of all exclusion criteria for Study 2 (left panel) and Study 3 (right panel). Note that responses were only possible after the stimulus disappeared (after 750 ms). The distribution of reaction times further supports that after applying the exclusion criteria subjects remaining in the sample were unlikely to have responded quickly and arbitrarily. Note that trials with very long reaction times (>15sec) were excluded from all analysis. **C.** Bar plots show histograms for the individual meta-d' values of the final sample in Study 2 (left panel) and Study 3 (right panel). For interpretation of metacognitive abilities in absolute terms, unconfounded by perceptual performance (d'), the ratio of meta-d'/d' is most useful. In study 2 the group average of meta-d'/d' was .75 (sd=.58) and in study 3 it was .79 (sd=.68). These distributions encompassed the theoretically optimal meta-d'/d' value of 1.

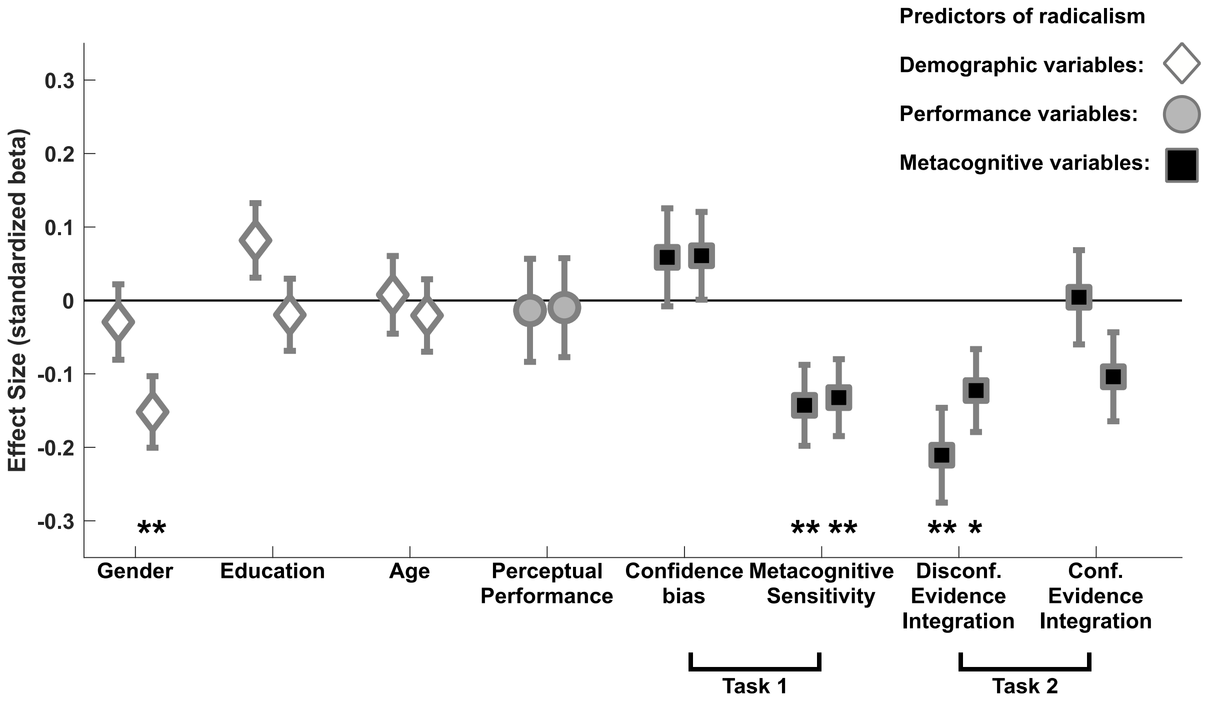

Figure S3. Impaired metacognitive sensitivity and reduced disconfirmatory evidence integration predict a composite measure of radicalism, related to Figure 3 & Figure 4. A composite measure of radicalism (obtained from the combined dogmatism and authoritarianism factor scores) was predicted by impaired metacognitive sensitivity and reduced disconfirmatory evidence integration, controlling for multiple demographic variables (gender, education, age) and other task-related variables (e.g. performance in the perceptual decision task and overconfidence bias). Here we present standardized beta coefficients ± standard error of predictors for Study 2 (left markers, N=381) and Study 3 (right markers, N=417). Effects in Study 3 were tested one-tailed based on the directional hypothesis derived from Study 2. Because we rewarded participants for accurate confidence ratings, the metacognitive failures of radicals led to reduced earnings compared to moderates (Study 2: $\beta=-.09$, $p=.008$; Study 3: $\beta=-.07$, one-tailed $p=.026$).   *$p<.05$; **$p<.01$. Task1 = Confidence task; Task 2 = Post-decision evidence integration task; Perceptual performance = Perceptual performance averaged across Task 1 and Task 2.

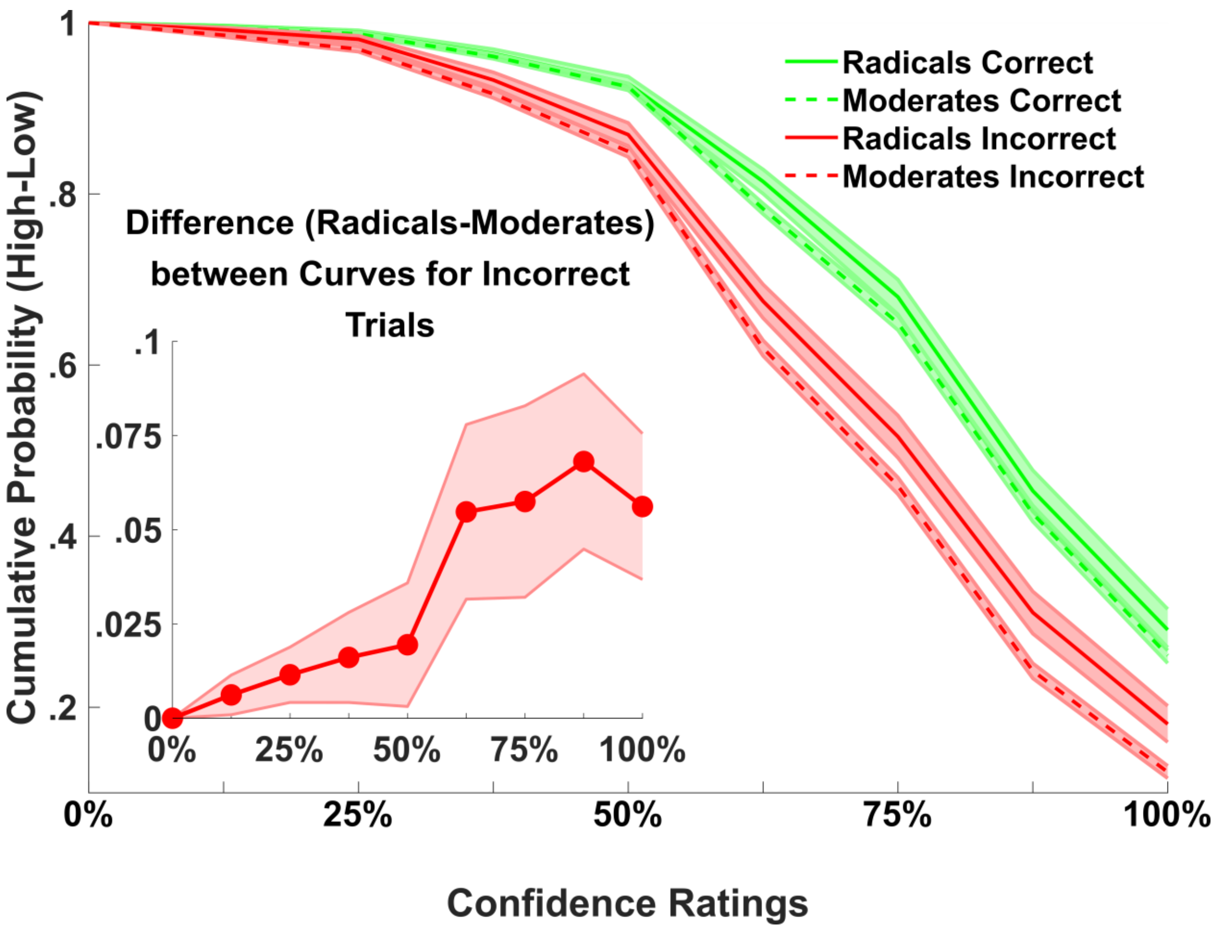

Figure S4. Reduced metacognitive sensitivity in radicals is driven by higher confidence in incorrect decisions, related to Figure 3 & Figure 4. The probability of choosing a particular confidence rating or a higher rating (cumulative probability from high to low) is presented for the 15 % most radical participants (radicals) and the rest of the sample (moderates), separately for correct and incorrect decisions. Here we present group averages ± standard error for data pooled from Study 2 and 3. A steep decline in the cumulative probability indicates that participants provide lower confidence ratings more frequently than high confidence ratings. The graph in the lower left panel shows the difference in cumulative probability between radicals and moderates on incorrect trials, indicating that radicals more frequently hold high confidence in their incorrect decisions than moderates.
